# Sustained release of cytokines from migrasomes

**DOI:** 10.20517/evcna.2025.15

**Published:** 2025-03-20

**Authors:** Yan Zhen, Harald Stenmark

**Affiliations:** ^1^Department of Molecular Cell Biology, Institute for Cancer Research, Oslo University Hospital, Oslo 0379, Norway.; ^2^Centre for Cancer Cell Reprogramming, Faculty of Medicine, University of Oslo, Oslo 0379, Norway.

**Keywords:** Cytokine, extracellular vesicles, inflammation, interleukin, migrasome, monocyte

## Abstract

Cytokines are released by cells in response to infections and tissue damage. A recent paper by Jiao *et al*. demonstrates that circulating monocytes release the cytokines tumor necrosis factor and Interleukin-6 encapsulated in large extracellular vesicles called migrasomes, from which the cytokines are secreted locally in a sustained fashion.

## MAIN TEXT

When cells in our body are exposed to insults such as infection or tissue damage, they transmit signals to immune cells that are recruited and activated to mediate inflammatory responses. Cytokines, small proteins released by many cell types, are key to such signaling. By binding to receptors on the source cells, neighboring cells, or distant cells, cytokines can perform both local and systemic activities. It has remained enigmatic how systemic effects of cytokines can be maintained since local cytokine gradients are known to be important for their action. This is particularly the case for cytokine-releasing cells in the circulation, since avid cell and fluid movement will preclude the buildup of cytokine gradients. A recent paper by Jiao *et al*. offers an explanation for how moving cells can cause local cytokine gradients in the bloodstream^[1]^.

The authors studied monocytes, large immune cells that circulate in the blood for a couple of days before entering tissues to differentiate into macrophages or antigen-presenting dendritic cells. Monocytes in the blood are known to be major sources of cytokine secretion in response to lipopolysaccharide (LPS), a compound found on the outer membrane of gram-negative bacteria, and Jiao *et al*. asked how these cells can induce local cytokine gradients in spite of their presence in the dynamic environment of the blood flow^[1]^.

A few years ago, Ma *et al*. made the seminal discovery that migrating cells leave behind a trail of large extracellular vesicles, migrasomes^[2]^. These vesicles develop from thin retraction fibers at the rear end of the migrating cell through mechanisms that require microscale domains of tetraspanin proteins and localized phosphoinositide signaling^[3,4]^. An interesting feature of migrasomes is that they are enriched in vesicles that contain signaling molecules, similar to the accumulation of synaptic vesicles in nerve terminals^[5]^, and migrating monocytes have been found to deposit migrasomes that contain the angiogenesis-inducing molecules VEGFA and CXCL12^[6]^. The authors, therefore, hypothesized that migrasomes released by monocytes might contribute to localized cytokine release. To test their hypothesis, they established a model that allowed the detection of monocytes in liver blood vessels of live mice using intravital microscopy. As expected, monocyte numbers increased sharply after intraperitoneal injection of LPS. Interestingly, the cytokine tumor necrosis factor (TNF) was detected in vesicles at the trailing end of these monocytes, and shedding of the vesicles could be demonstrated by time-lapse imaging. Isolation and analyses of the shed vesicles showed that they shared the morphological characteristics of migrasomes, including their attachment to retraction fibers, and that they contained TNF, as well as another potent cytokine, interleukin-6 (IL-6).

A rise in cytosolic calcium concentration is known to trigger cytokine release^[7]^, and Jiao *et al*. used a calcium blocker to prevent cytokine release from isolated monocytes^[1]^. Interestingly, the calcium blocker also inhibited cytokine release from isolated migrasomes, and it led to a more than 10-fold increase in the cytokine content of the migrasomes, whereas the total cellular content of cytokines only increased by about 40%. This indicates that migrasomes are a major source of cytokine release from monocytes. Consistent with this, electron microscopy revealed the presence of numerous small vesicles in migrasomes, and these were found to contain Rab GTPases^[8]^ and SNARE proteins^[9]^ known to be involved in exocytosis^[1]^. Treatment with calcium blockers increased the numbers of these vesicles, as expected if they are released by calcium-dependent exocytosis.

Time-lapse imaging of migrating monocytes on fibronectin-covered surfaces showed a strong enrichment of TNF and IL-6-containing vesicles in the rear end of the cells, and some of these could be seen entering migrasomes. If cell migration was inhibited, this polarized localization was lost, supporting the notion that cell migration causes polarization of cytokine-containing exocytic vesicle to the cell rear, where they enter into migrasomes.

Previous work has indicated that tetraspanin proteins are involved in migrasome biogenesis^[3]^, and knockout of the tetraspanin Tspan9 inhibits migrasome biogenesis in mice^[10]^. In line with this, migrasome formation was reduced in monocytes derived from Tspan9 knockout mice, and this was accompanied by decreased release of TNF and IL-6^[1]^. Using imaging flow cytometry of whole blood with antibodies against CCR2, a chemokine receptor specifically found on monocyte membranes, Jiao *et al.* observed that blood from Tspan9 knockout mice contained significantly less CCR2-positive extracellular vesicles than blood from wild-type mice^[1]^. Biochemical analyses confirmed that these vesicles represented migrasomes and not other types of extracellular vesicles. When monocyte-derived migrasomes were isolated from mouse blood, it was estimated that about 50% of TNF and 24% of IL-6 were secreted in association with migrasomes, and secretion was significantly reduced in blood from Tspan9 knockout mice. This provides compelling evidence that migrasomes serve as major mediators of TNF and IL-6 release from migrating monocytes in the blood.

To address whether isolated migrasomes can indeed release cytokines, Jiao *et al*. isolated detached migrasomes from cells expressing fluorescently tagged TNF and studied them by time-lapse fluorescence microscopy^[1]^. They noticed that TNF-positive vesicles progressively fused with the limiting membrane of the migrasomes in calcium-containing medium, providing evidence for cytokine release from detached migrasomes. Likewise, release of TNF and IL-6 could be detected from migrasomes isolated from serum after incubation in calcium-containing medium, and this release was inhibited by a calcium blocker.

**Figure 1:**
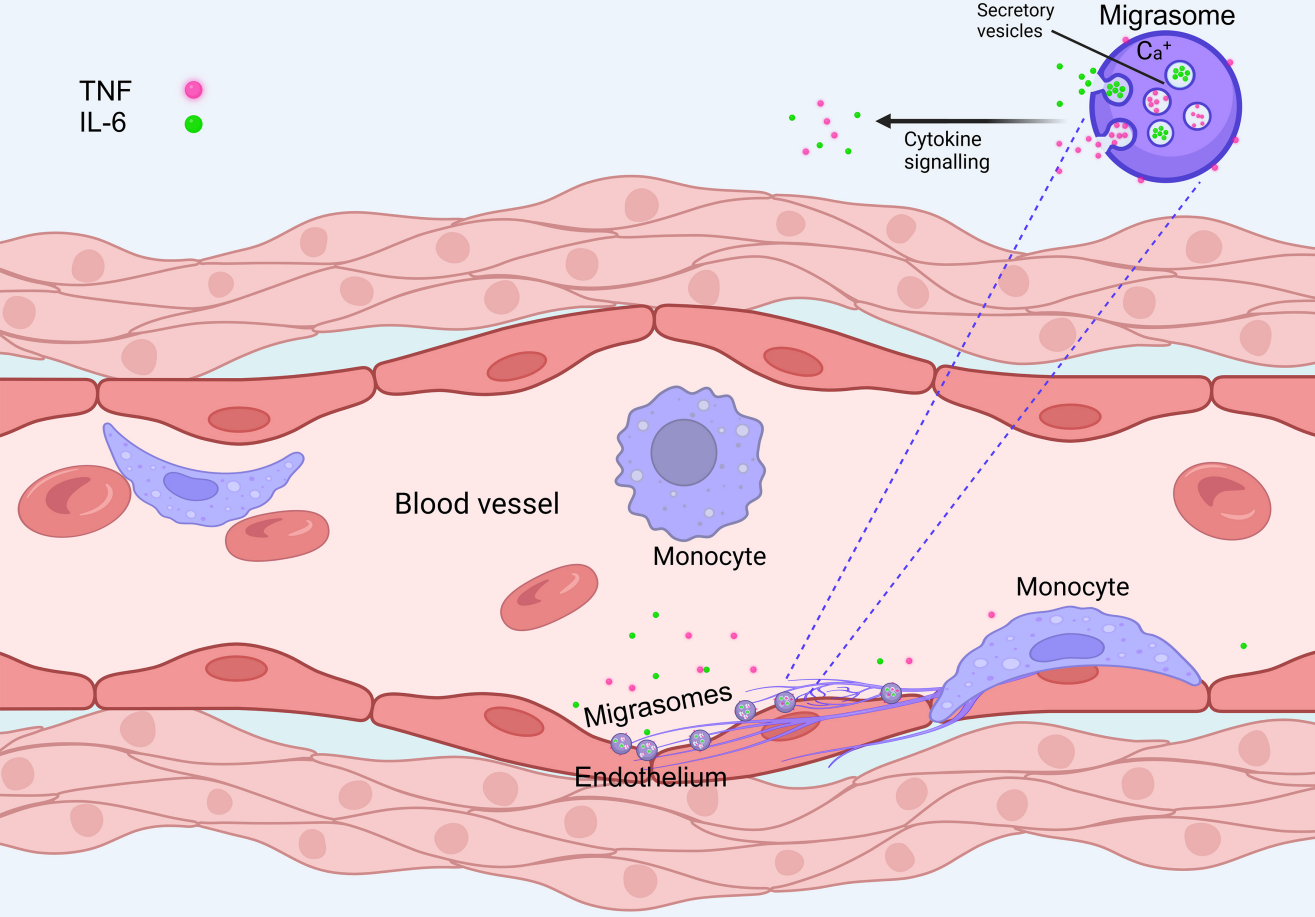
Adherent monocytes within blood vessels release migrasomes at their rear end during migration. The migrasomes accumulate secretory vesicles that contain the cytokines IL-6 and TNF. When exposed to calcium signaling, the vesicles fuse with the limiting membrane of the migrasome, causing local release of cytokines at the sites where migrasomes are deposited. TNF exists in both soluble and membrane-bound form, and the membrane-bound form remains in the limiting membrane of the migrasomes. Figure prepared with biorender.com. IL-6: Interleukin-6; TNF: tumor necrosis factor.

TNF is produced in cells as a transmembrane protein, which can be cleaved by the protease tumor necrosis factor converting enzyme (TACE) to release the extracellular part^[11]^. Both forms can transmit signaling, and Jiao *et al*. found that migrasomes contain both processed and unprocessed TNF^[1]^. Moreover, they found that monocyte-derived migrasomes in combination with a caspase-8 inhibitor could induce necroptosis in a well-described assay for TNF activity, and this effect was partially reduced in the presence of a TACE inhibitor. Taken together, these studies indicate that detached migrasomes can function as sustained-release agents for cytokines [Figure 1].

The “advantage” of migrasome-mediated release of cytokines is that sustained cytokine release can be maintained at restricted sites even in the presence of blood flow. This was demonstrated by the finding that monocyte-derived migrasomes accumulated at sites of LPS injection in mice, and knockout of Tspan9 strongly inhibited this effect. Thus, monocyte-derived migrasomes can function as sustained local sources of cytokine release during inflammatory responses.

The novel findings raise several intriguing perspectives, including the fact that migrasomes can be produced by many migrating cell types^[12]^. It will be interesting to learn whether additional cytokines are released by monocyte-derived migrasomes, and to what extent, for instance, circulating neutrophils and tumor cells also use migrasomes for creating sustained signaling. If so, this could have therapeutic implications. For the physiological role of migrasomes, it will be important to study knockout mice with additional defects in migrasome biogenesis, given that the knockout of Tspan9 might affect additional functions beyond migrasome biogenesis, including cell signaling and motility. While the release of cytokines from migrasomes is clearly calcium-dependent, it will be important to investigate whether calcium signaling is regulated in migrasomes, and whether external stimuli other than calcium can trigger their cytokine release.

## DECLARATIONS

### Copyright

© The Author(s) 2025.
